# Deregulation of Notch1 pathway and circulating endothelial progenitor cell (EPC) number in patients with bicuspid aortic valve with and without ascending aorta aneurysm

**DOI:** 10.1038/s41598-018-32170-2

**Published:** 2018-09-14

**Authors:** Carmela R. Balistreri, Floriana Crapanzano, Leonardo Schirone, Alberto Allegra, Calogera Pisano, Giovanni Ruvolo, Maurizio Forte, Ernesto Greco, Elena Cavarretta, Antonino G. M. Marullo, Sebastiano Sciarretta, Giacomo Frati

**Affiliations:** 1 Department of Pathobiology and Medical Biotechnologies, University of Palermo, Palermo, Italy; 2 Department of Medico-Surgical Sciences and Biotechnologies, Sapienza University of Rome, Latina, Italy; 3 Department of Surgery and Oncology, University of Palermo, Palermo, Italy; 4 Department of Experimental Medicine and Surgery, University of Rome Tor Vergata, Rome, Italy; 5 IRCCS NEUROMED, Pozzilli, IS Italy; 6 Department of Cardiovascular, Respiratory, Nephrological, Anesthesiological, and Geriatric Sciences, Sapienza University of Rome, Rome, Italy

## Abstract

Bicuspid aortic valve (BAV) is frequently associated with the development of ascending aortic aneurysm, even if the underlying mechanisms remain to be clarified. Here, we investigated if a deregulation of Notch1 signaling pathway and endothelial progenitor cells (EPCs) number is associated with BAV disease and an early ascending aortic aneurysm (AAA) onset. For this purpose, 70 subjects with BAV (M/F 50/20; mean age: 58.8 ± 14.8 years) and 70 subjects with tricuspid aortic valve (TAV) (M/F 35/35; mean age: 69.1 ± 12.8 years) and AAA complicated or not, were included. Interestingly, patients with AAA showed a significant increase in circulating Notch1 levels and EPC number than subjects without AAA. However, circulating Notch1 levels and EPC number were significantly lower in BAV subjects than TAV patients either in the presence or absence of AAA. Finally, Notch pathway was activated to a greater extent in aortic aneurysmatic portions with respect to healthy aortic fragments in both BAV and TAV patients. However, the expression of genes encoding components and ligands of Notch pathway in aortic tissues was significantly lower in BAV than TAV subjects. Our study demonstrates that BAV subjects are characterized by a significant decrease in both tissue and circulating levels of Notch pathway, and in blood EPC number than TAV patients, either in presence or absence of AAA disease.

## Introduction

Bicuspid aortic valve (BAV) is the most common congenital cardiac malformation, affecting approximately 1.3% of the population worldwide^1^. It may evolve into a non-homogeneous leaflet calcification and serious complications, occurring in about 33% of patients^1–3^. Among the serious complications, ascending aortic aneurysm (AAA) and dissection are frequent^1^. Aortic aneurysm development and rupture associated with BAV are likely the results of anomalous regulatory pathways in endothelial cells and vascular smooth muscle cells within the aortic media. Unfortunately, these mechanisms remain to be fully clarified. The elucidation of this aspect appears to be particularly important, since it may help to find new therapeutic targets for the prevention of the development and progression of AAA.

Accumulating lines of evidence demonstrated that an impairment of Notch signaling pathway is involved in the development and progression of aortic aneurysm, showing a dual effect^4–6^. On the one hand, Notch1 appears to be activated in aortic aneurysms and either genetic or pharmacological attenuation of this pathway delays aneurysm progression^6–9^. On the other hand, the effects of Notch1 activation appears to be cell-dependent, and it was previously shown that Notch pathway is critical for the maintenance of vascular integrity and promotion of vascular repair^4–6,10–14^. Accordingly, a deregulation of Notch signaling pathway may be involved in the development of vascular complications associated with BAV, since previous works showed that a mutation of Notch1 gene sequence is frequently associated in subjects with BAV^15,^^16^. However, a direct comparison of the level of systemic and vascular activation of Notch signaling pathway between patients with TAV and BAV has never been performed.

Notch signaling pathway plays critical functions in the regulation of new vessel formation, neo-angiogenesis and vascular repair during stress^6,14–16^. These vascular functions controlled by Notch signaling pathway may partially involve the regulation of endothelial progenitor cells (EPC). In fact, it was previously demonstrated that Notch signaling pathway promotes EPC differentiation and mobilization^17,18^. Interestingly, a reduction of EPC number and function was previously found to be associated with the formation of cerebral and aortic aneurysm and inversely correlated with the size of aortic aneurysms^19,20^. These data suggest that an impairment of EPC system is associated with derangements in vascular homeostasis and finally with the formation of vascular structure abnormalities.

Thus, we hypothesized that a deregulation of Notch1 signaling pathway and EPC number is associated with the presence of BAV, thereby early contributing to the development of AAA. Accordingly, the aim of our study was to investigate the interplay of Notch signaling pathway activity with EPC number, in BAV and AAA pathologies in a cohort of patients with BAV or TAV.

## Results

### BAV and TAV patient characteristics

BAV and TAV patient’s features are summarized in Table 1. No significant differences were observed regarding the size of aorta dilatation between BAV and TAV cases affected by AAA. However, many BAV cases were affected by AAA. Specifically, of 70 BAV cases, 51 subjects (73%; 45 males and 6 females; mean age: 50.6 ± 16.6 years) also showed AAA with respect to only 25 TAV cases affected by AAA (36%; 19 males and 6 females; mean age: 70.2 ± 8.9 years) (p = 0.00001, by *χ*2 test, respectively). However, in both two groups of cases, the aneurysm had a location in the tubular ascending aorta portion. Among the 51 BAV patients with AAA, 35 (69%; stenotic or incontinent) showed a fibrocalcific valve and 8 (16%) a prolapsed valve (Table 1). The remaining 19 BAV subjects (27%; 5 males and 14 females; mean age: 57.38 ± 15.3 years) were not affected by AAA, even if they presented aortic valve stenosis (73%) and/or a fibrocalcific aortic valve (22%). In addition, the 25 TAV cases with AAA had a moderate valve incontinence and stenosis. The remaining 45 TAV subjects (64%; 16 males and 29 females; mean age: 66.2 ± 13.3 years) without AAA showed valve stenosis only in 7 subjects (15%; 4 males and 2 females; mean age: 69.5 ± 12.4 years).

**Table 1:** Demographic and clinical characteristics, comorbodity conditions, complications of 70 BAV and 70 TAV subjects.

Variables		BAV N = 70	TAV N = 70	P
***Demographic characteristics***	Age, mean (SD)	58.8 (14.8)	69.1(12.8)	<0.0001
	Male sex, No. (%)*	50 (71)	35(50)	0.009
	Female sex, No. (%)	20 (29)	35 (50)	0.009
	Body mass index, mean (SD)	26 (4.8)	26.3 (3.2)	N.S.
***Size and location of AAA***	Subjects affected (%)	51 (73)	25(36)	0.00001
	Size (mm), mean (SD)	53.3 (7.4)	50.3 (6.9)	N.S.
	Location, No:			
	Tubular ascending aorta	51 (100)	25 (100)	N.S.
***Comorbidity conditions, No (%)***	CVD Family History	8 (11)	5 (7.1)	N.S.
	Smoking	26 (37)	20 (28)	N.S.
	Hypertension	55 (78)	50 (719	N.S.
	Dyslipidemia	9 (13)	5 (7.1)	N.S.
	Diabetes mellitus	3 (4.2)	1 (1.4)	N.S.
	Renal failure	0 (0)	1 (1.4)	N.S.
	Dissection	0 (0)	0 (0)	N.S.
***Aortic valve pathology, No (%):***				
	Normal	0 (0)	38 (54)	0.0001
	Prolapse	8 (11)	0(0)	0.003
	Vascular calcium fibrosis	35 (50)	7 (10)	2.4e-7
***Atherosclerosis coronary syndrome, No(%):***		2 (3)	1 (0.8)	N.S.

^*^Percentage values on total BAV and TAV subjects.

*P* = *TAV vs. BAV, by t Student test for quantitative variables, or χ*^*2*^ *test for qualitative variables*.

### Circulating number of the four EPC populations in BAV vs. TAV groups

We examined whether there were significant differences in the circulating levels of EPC cell populations between the BAV and TAV groups. Four different populations of EPCs were quantified: (1) CD45^dim^CD133^+^KDR^+^ cells; (2) CD45^dim^CD34^+^KDR^+^cells; (3) CD45^dim^CD34^+^CD133^+^KDR^+^triple positive cells; and 4) the subpopulation of CD45^dim^CD34^+^KDR^+^CXCR4^+^ EPCs. These four subtypes represent EPCs with a different level of maturation (see Materials and Methods Section).

**Figure 1:**
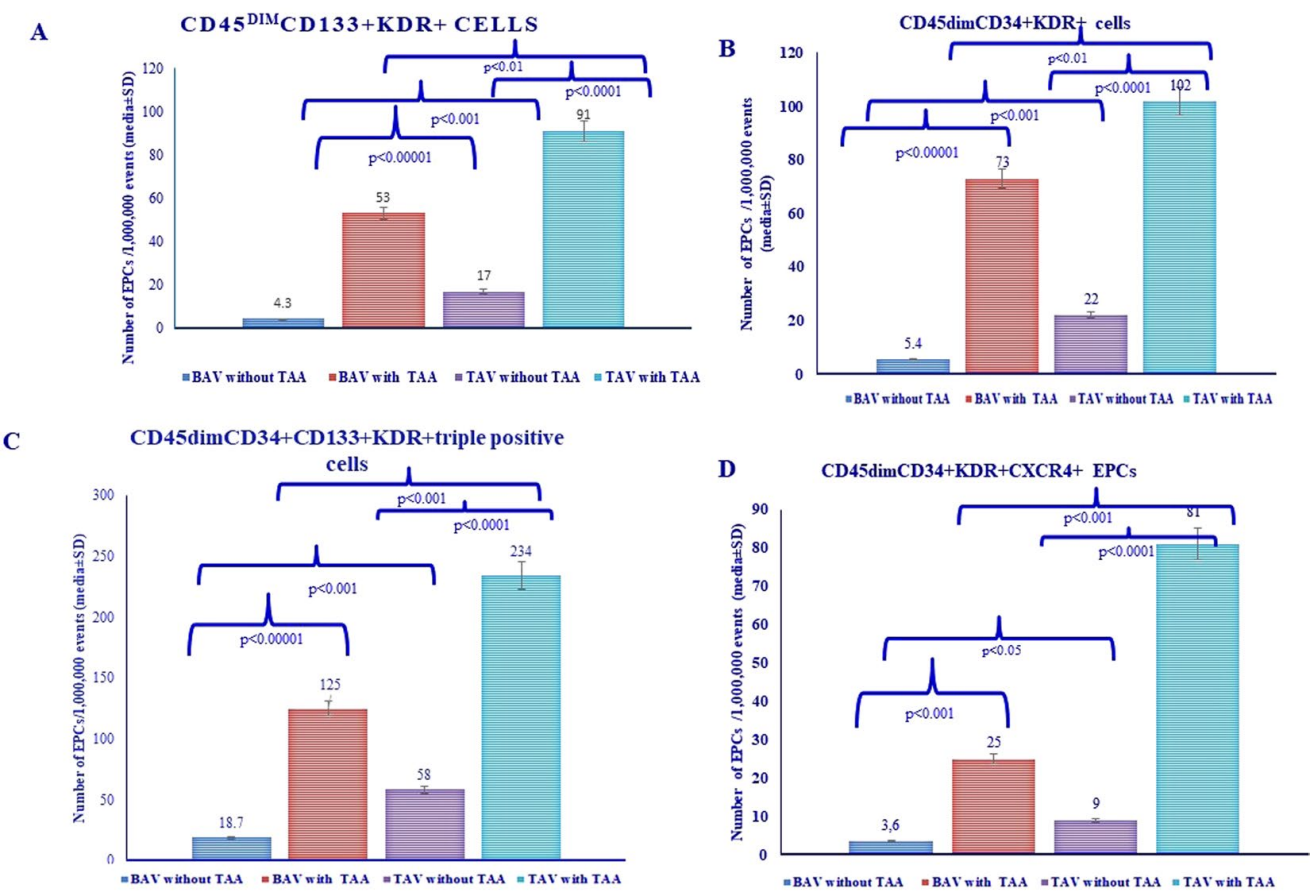
Circulating levels of the four EPC populations detected in peripheral blood from the study population. (**A**–**D**) Circulating number of different EPC subpopulations was measured in different patient groups. Immunophenotype of the four EPC populations is displayed in the Figure. Of note, population 1 was CD34-negative, whereas population 2 was CD133-negative.

As shown in Fig. 1A–D, the circulating number of all four EPC cell populations showed similar differences in diverse patient’s groups. Patients with BAV without AAA displayed the lowest circulating number of four EPC populations, which was significantly lower than that observed in TAV without AAA. Interestingly, both TAV and BAV groups with AAA showed a significant elevation in the number of the four EPC populations (see Fig. 1A–D) with respect to TAV and BAV groups without AAA. However, among patients with AAA, the circulating number of EPCs in BAV subjects was still significantly lower than that observed in TAV subjects. Of note, the slope of increase of immature EPC (population 1 and 2) in patients with AAA with respect to those without AAA tended to be higher in patients with BAV than those with TAV. However, no changes were observed regarding population 3 and an opposite trend was found with regard to population 4, which represented the most mature form of EPC. Overall, these results may suggest that formation of EPC is boosted in patients with BAV and AAA as a compensatory mechanism for the reduction of their circulating number and for the presence of AAA. However, the maturation process of these cells is impaired in these patients leading to a lower yield of mature EPC.

### Systemic plasma levels of Notch1, SDF-1, and VEGF molecules

The low levels of the four circulating EPC populations detected in BAV groups led us to investigate eventual dysfunctions in the molecules involved in EPC physiological function and maturation, such as Notch 1 pathway, SDF-1, and VEGF molecules. Consequently, we first assessed the systemic levels of these molecules (see Table 2). Regarding Notch 1 pathway, we evaluated the levels of the soluble form, deriving from an alternative way of activation of Notch 1 pathway, which occurs in both physiological and pathological conditions. This way is principally mediated by several factors, such as proteins secreted by endothelial and smooth muscle cells, and it determines the dissociation of Notch 1 extracellular domain and receptor activation^21–24^. Thus, we assessed the circulating levels of Notch soluble form in both BAV and TAV cases. Surprisingly, the comparisons of the data obtained on systemic levels of Notch1 highlighted that BAV cases without AAA show the lowest levels of Notch1 with respect to non-complicated TAV individuals (see Table 2), suggesting a deregulation of this pathway. In addition, among patients with AAA, individuals with BAV also had significantly reduced plasma levels of Notch1 when compared to TAV subjects (Table 2). Furthermore, comparisons of plasma levels of VEGF and SDF-1 in the groups enrolled, which are known to play an important role in angiogenesis by recruiting endothelial progenitor cells, were also assessed. We found that the levels of these molecules were lower in BAV without AAA patients than those detected in TAV without AAA subjects. However, no differences were observed between BAV and TAV groups affected by AAA (see Table 2). Thus, we excluded that the reduction of EPC levels in BAV groups with AAA was secondary to a deregulation of VEGF and SDF-1 circulating levels.

**Table 2:** Systemic plasma levels of Notch1, SDF-1, and VEGF.

Systemic molecules examined	BAV without TAA N = 19	BAV with TAA N = 51	P
**Notch-1 (pg/ml)**	0.05 ± 3	11.3 ± 6	**<0.0001**
**SDF-1 (pg/ml)**	1256.2 ± 8	1998.2 ± 15	**<0.0001**
**VEGF (pg/ml)**	293 ± 13	408 ± 16	**<0.0001**
**Systemic molecules examined**	**TAV without TAA N** = **45**	**TAV with TAA N** = **25**	**P**
**Notch-1 (pg/ml)**	6.9 ± 3	21.6 ± 8	**<0.0001**
**SDF-1 (pg/ml)**	1658.8 ± 11	1995 ± 1	**<0.001**
**VEGF (pg/ml)**	389 ± 7	405 ± 2	**<0.001**
**Systemic molecules examined**	**BAV without TAA N** = **19**	**TAV without TAA N** = **45**	**P**
**Notch-1 (pg/ml)**	0.05 ± 3	6.9 ± 3	**<0.0001**
**SDF-1 (pg/ml)**	1256.2 ± 8	1658.8 ± 11	**<0.001**
**VEGF (pg/ml)**	293 ± 13	389 ± 7	**<0.001**
**Systemic molecules examined**	**BAV with TAA N** = **51**	**TAV with TAA N** = **25**	**P**
**Notch-1 (pg/ml)**	11.3 ± 6	21.6 ± 8	**<0.0001**
**SDF-1 (pg/ml)**	1998.2 ± 15	1995 ± 1	0.08
**VEGF (pg/ml)**	408 ± 16	405 ± 2	0.19

*Vascular endothelial growth factor (VEGF), Stromal cell-derived factor- (SDF)-1*.

*By unpaired t-test with Welch correction.

### Correlations between the four EPC cell populations and systemic factors

**Figure 2:**
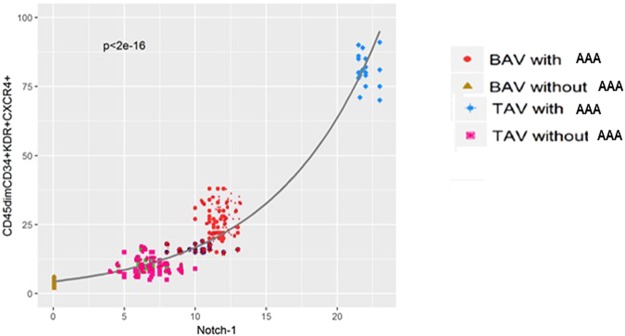
Linear relationship between Notch-1 and CD45dimCD34+KDR+CXCR4+EPCs. Generalized linear model with Poisson distribution and logarithm link correlating circulating Notch1 soluble levels with EPC subpopulation. Statistical significance tested among all groups (P < 2^E-16^).

The data obtained on systemic molecules led us to determine which circulating factor was the strongest predictor for EPC number in our population. Consequently, we determined the potential correlations between the four EPC populations and Notch 1, SDF-1, and VEGF molecules. Interestingly, we detected that the reduction of all the four EPC cell populations observed in BAV cases positively correlated only with the Notch 1 soluble molecule (r = 0.131, p = 0.01; r = 0.229, p = 0.02; r = 0.267, p = 0.001, r = 0.345, p = 0.0001, respectively; by non-parametrical Spearman correlation test; data not shown). This data was additionally confirmed by a generalized linear model with Poisson distribution and logarithm link. It showed that Notch1 was the only factor to be significantly associated with the three most relevant and functional EPC subpopulations, and particularly with the CD45^dim^CD34+KDR+CXCR4+EPC population (the most differentiated EPC population) (p < 2^e-16^; see Fig. 2A). In addition, by simply comparing the values of different circulating factors with the number of CD45^dim^CD34+KDR+CXCR4+EPC cells on the same graph, differences in Notch1 levels and EPC number among different groups showed the same trend (see Table 3).

**Table 3:** Simple comparison of amounts of circulating angiogenetic factors, Notch1, and the triple positive EPC cells.

Amounts of circulating angiogenetic factors, Notch1 and the triple positive EPC cells	BAV without TAA N = 19	BAV with TAA N = 51	TAV without TAA N = 45	TAV with TAA N = 25
**CD45** ^**dim**^ **CD34 + KDR + CXCR4 + EPCs**	3.6 ± 1.2	25 ± 2.5	9 ± 3.4	81 ± 6.4
**Notch-1 (pg/ml)**	0.05 ± 3	11.3 ± 6	6.9 ± 3	21.6 ± 8
**SDF-1 (pg/ml)**	1256.2 ± 8	1998.2 ± 15	1658.8 ± 11	1995 ± 1
**VEGF (pg/ml)**	293 ± 13	408 ± 16	389 ± 7	405 ± 2

### Gene expression levels of Notch signaling components in aortic tissues from BAV and TAV patients and their tissue protein levels

**Figure 3:**
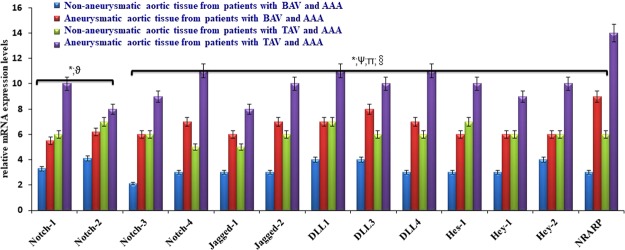
Expression levels of Notch signaling in aortic aneurysms. Significant differences were observed among the groups with the lowest levels detected in BAV groups than TAV cases and controls (see details in Result section). The statistical significance was tested as it follows: aneurysmatic aortic tissue from patients with BAV and AAA vs. aneurysmatic aortic tissue from patients with TAV and AAA (*P < 0.001); non-aneurysmatic aortic tissue from patients with TAV and AAA vs. aneurysmatic aortic tissue from patients with TAV and AAA(^ϑ^P < 0.05); non-aneurysmatic aortic tissue from patients with TAV and AAA vs. aneurysmatic aortic tissue from patients with TAV and AAA (^ψ^P < 0.001); non-aneurysmatic aortic tissue from patients with BAV and AAA vs. aneurysmatic aortic tissue from patients with BAV and AAA (^§^P < 0.001); non-aneurysmatic aortic tissue from patients with BAV and AAA vs. non-aneurysmatic aortic tissue from patients with TAV and AAA (^π^P < 0.001).

To validate the significant reduction of levels of Notch1 in plasma samples of BAV cases, we also checked Notch signaling pathway in aortic tissues from patients with BAV and TAV affected by AAA undergoing surgery. We indeed evaluated the gene expression levels of all Notch receptors (1–4), Notch ligands and Notch downstream targets (such as the Hairy and enhancer of split gene family (Hes), the Hes related gene family (Hey) and the Notch-regulated ankyrin repeat protein (NRARP)) in portions of normal aorta and portions of aortic aneurysm harvested from patients underwent elective surgical repair. As shown in Fig. 3, all genes belonging to Notch signaling showed the same expression pattern. Notch signaling was activated in aneurysmatic portions with respect to aortic healthy tracts in both BAV and TAV patients. However, Notch expression levels were significantly lower in both aneurysms and normal aortic parts from subjects with BAV with respect to patients with TAV, confirming the previously described results obtained with circulating levels of soluble Notch1.

## Discussion

Over the last couple of years, it has become increasingly evident that the Notch signaling pathway plays a pivotal role in the development and homeostasis of the cardiovascular system. The Notch signaling pathway is critical for cell fate determination during embryonic development, including many aspects of vascular development. An emerging paradigm suggests that the Notch gene regulatory network is often involved in vascular remodeling and repair in the context of several vascular diseases following injury.

Our study demonstrates that Notch signaling is activated in patients affected by AAA, both in the peripheral blood as well as in specimens of aneurysmatic tissues. These results were paralleled by an increase in the number of circulating EPCs in these subjects. Interestingly, Notch signaling activity and EPC number were significantly reduced in patients affected by BAV either in the presence or in the absence of AAA. Overall, these data suggest that a deregulation of Notch1 and EPC may be implicated in the development of BAV and the associated vascular complications.

Previous study demonstrated that derangements of Notch signaling are involved in the development of aortic aneurysms^7–9^. Notch signaling consists of four receptors (Notch1–4), with either overlapping or distinct functions, which bind to specific ligands (Jagged1/2; Delta-like1/3/4)^4–6^. Notch receptors and ligands are highly expressed in the vasculature, showing some differences in their expression pattern among different vascular cells and playing both physiological and pathological functions^4–6^. Notch signaling regulates vasculogenesis and endothelial mesenchymal transition during development^4–6,11,12^. Notch signaling was also shown to preserve vascular integrity and to promote the formation of new vessels and vascular repair in response to stress^10–14^. Previous studies also demonstrated that Notch signaling components and precisely the extracellular domain can be found in circulating soluble forms eliciting receptor activation in a ligand-independent manner^21–24^.

Our study significantly extends this previous evidence by demonstrating that the entire Notch signaling pathway is activated in thoracic aortic aneurysms with respect to normal aortic tissues. However, the aortic gene expression of all Notch receptors and ligands was found to be reduced in patients affected by BAV. BAV patients with and without AAA also displayed a reduction in circulating levels of soluble Notch1 with respect to patients with TAV. Circulating Notch1 levels were also significantly lower in patients with BAV without AAA. These data are in agreement with previous study demonstrating that BAV condition is associated with genetic defects of Notch1^15,16^ and may suggest that an impairment of the entire Notch signaling may contribute to the development of aortic complications in patients affected by BAV. Certainly, future mechanistic studies are mandatory to corroborate this hypothesis. Of note, since Notch1 signaling deeply relies on the tissue microenvironment, future studies are necessary to evaluate whether these mechanisms may be involved in the inhibition of Notch1 signaling in patients with BAV. It is possible that several mechanisms and interplays related to extracellular matrix and connective tissue remodeling in patients with BAV may contribute to the observed derangements. Genetic mechanisms may also be involved in the impairment of Notch1 signaling in patients with BAV. In fact, previous work showed that a mutation of Notch1 gene sequence is frequently associated in subjects with BAV^15,16^.

We also found that the reduction of circulating levels of soluble Notch1 in subjects affected by BAV are directly correlated with the reduction of circulating EPC number observed in these subjects. This result is in line with previous studies demonstrating that activation of Notch signaling pathway contributes to EPC mobilization and differentiation during stress^15,17^. EPCs play a critical role in vascular repair processes during stress^25–27^. Accordingly, previous study demonstrated that an impairment of EPCs is associated with the development of cerebral and aortic aneurysms^19,20^. Indeed, it was demonstrated that the number of circulating EPCs in subjects with aortic aneurysm is inversely correlated with the severity of the disease^19^. This evidence may support the hypothesis that a deregulation of the Notch/EPC axis may represent a potential mechanism through which BAV condition is associated with an increased susceptibility to develop pathological aortic diseases. Future experimental studies are warranted to investigate the mechanisms underlying the impairment of Notch signaling and EPC in subjects with BAV.

### Strengths and limitations

The strengths of the present study include the homogenous study population and the very comprehensive data regarding the population enrolled. Moreover, the data of this study are the result of a relatively small sample, since we focused our study on BAV, a disease with a very low prevalence. Nevertheless, our data are relevant, even if they need additional validation and confirmation. However, our study has limitations that should be acknowledged. The main limitation of the study relies on the fact that it was associative, and we could not appropriately study the cause-effect relationship between Notch signaling, EPC, aortic aneurysm and BAV. Another limitation could be represented by the fact that age was lower in subjects with BAV thereby hypothetically interfering with the results. In fact, this depends on the early onset of complications in BAV cases than TAV cases. BAV should be considered a heterogeneous and multifaceted disease, with a great clinical impact, being associated with the onset of a large range of serious diseases, such as AAA and aortic dissection. Furthermore, we could not corroborate gene expression analyses in aortic specimens by checking also protein expression of different genes. Consequently, further and larger investigations are certainly needed. For example, genomic, transcriptomic and epigenetic investigations may eventually lead to a better understanding of the molecular and cellular mechanisms associated with AAA in BAV patients^28^.

## Conclusions

**Figure 4:**
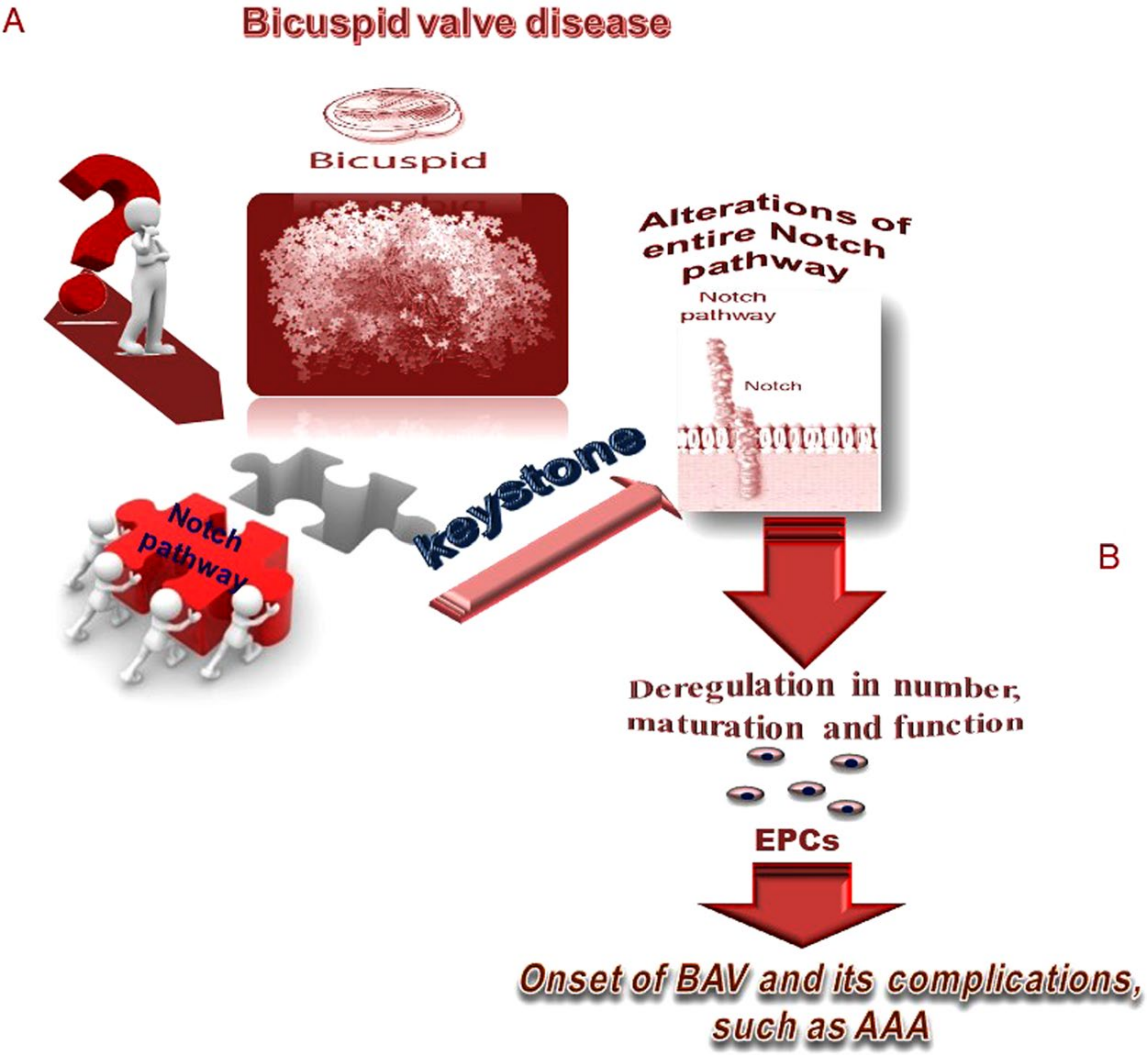
(**A**,**B**) Our model on the pathophysiological mechanisms underlying the development of BAV and its vascular complications, the *model from funny to Eureka*. In (**A**) we suggest that BAV is the result of alterations in Notch pathway, which determine a deregulation in both number and function of EPC cells, and consequently in the cardiovascular repair system. In (**B**) we evidence that all these alterations early induce vascular dysfunction and remodelling and consequently onset of AAA.

In conclusion, our study demonstrates that subjects with BAV have a significant reduction of Notch signaling and EPC number with respect to patients with TAV, irrespective to the presence of AAA, suggesting a deregulation in Notch signaling and EPC in subjects with BAV (a suggested model is reported in Fig. 4A,B, defined as “*model from funny to Eureka”*). Furthermore, the results of this study confirm the data previously obtained in our recent investigations^29–31^, which suggest the presence of unique molecular and cellular mechanisms in AAA development in BAV vs. TAV patients. Further and larger studies are certainly necessary to validate these findings and our suppositions. The reproducibility of our results from a very large sample size, enriched by clinical data, might provide the possibility of identifying potential molecular and genetic biomarkers useful to detect BAV subjects at high AAA risk, to monitor and treat them differently from those with TAV^21,30–32^. Thus, confirmation and validation of these emergent data could allow us to suggest specific clinical recommendations on the correct timing as well as the most appropriate surgical approach to use in the case of BAV patients with AAA and a normal aortic root in order to prevent or attenuate multiple possible complications, such as rupture or cerebral and peripheral adverse events. On the other hand, clarifying the issue concerning the surgical procedure with or without composite aortic root replacement represents the major aims of recent studies, since no clear and comprehensive guidelines do currently exist. Furthermore, as mentioned above, this approach could allow us to identify a multi-biomarker profile to facilitate the management and outcome of this complex syndrome.

## Material and Methods

### Study population

Our study included a population of 70 BAV subjects (50 males and 20 females; mean age: 58.8 ± 14.8 years) and 70 TAV subjects (35 males and 35 females; mean age: 69.1 ± 12.8 years) with or without AAA, as shown in Table 1. Patients of each group (precisely BAV with or without AAA, TAV with or without AAA) were recruited from January 2015 to December 2016. The cases were randomly selected from patients referring to the Units of Cardiac Surgery (Department of Surgery and Oncology, University of Palermo) and Cardiology, for surgery replacement or routine care screening. Appropriate exclusion criteria were also used during the BAV/TAV enrollment, for the following diseases: (a) cardiovascular diseases were excluded according to history and by detecting apposite laboratory and imaging biomarkers as indicated by the latest ESC or ASC guidelines; (b) connective tissue disorders were excluded by assessing markers of inflammation immunological (i.e. autoantibodies) and imaging biomarkers; (c) inflammatory diseases (from infections to hematological, gastrointestinal, urogenital, pulmonary, neurological, endocrinal inflammatory disorders, and neoplasies included) by detecting apposite laboratory parameters (including complete blood cell count, erythrocyte sedimentation rate, glucose, urea nitrogen, creatinine, electrolytes, C reactive protein, liver function tests, iron, and proteins) and imaging biomarkers. In addition, all the enrolled cases belonged to the same ethnic group, since their parents and grandparents were born in Western Sicily. Thus, a very homogenous population was studied. Furthermore, elective or acute surgical treatment (using wheat operation, Bentall-De Bono and Tirone David surgical techniques, whenever possible) and complementary tubular-ascending aorta resection were performed in the BAV and TAV patients with AAA after evaluation of aortic transverse diameter sizes by Computed Tomography scanning according to recent guidelines, as reported in our review^31,33^. Accordingly, an experienced physician evaluated aortic transverse diameter sizes by echocardiography (Philips Ie. 33) before either elective or urgent surgery. The dimension of the aortic annulus, sinuses of Valsalva, proximal ascending aorta (above 2.5 cm of the sino-tubular junction) and aortic arch were assessed pre-operatively by trans-thoracic echocardiography as well as in the operating theatre by trans-oesophageal-echocardiography before the institution of the cardiopulmonary bypass. These measures, together with demographic and clinical data (including co-morbidities) were obtained from patients’ medical records and are presented in Table 1. In all BAV and TAV cases, hypertension was treated by beta-blockers.

### Ethical Study Approval

Our study was performed in accordance with ethical standards of the Helsinki Declaration of the World Medical Association and Italian legislation, and it received approval from Regional Ethics Board in Palermo (No. APUNIP0094517) and all participants gave their informed consent. Data were encrypted to ensure patient s’ privacy. All clinical measurements were performed in blind.

### Flow cytometry quantification of the four EPC populations number

For the identification and quantification of EPCs, we used a slightly modified version of a protocol proposed for the first time in 2010 by Schmidt-Lucke and co-workers^34^ and based on the International Society for Hematotherapy and Graft Engineering (ISHAGE) sequential gating strategy^35^ (see details in Supplementary Material). Briefly, 1 ml of blood was collected from a forearm vein into EDTA-coated tubes, transported in ice to the laboratory and processed within 1 to 2 hours after the collection. First of all, 150 μl of whole blood were incubated with the following combination of anti-human monoclonal antibodies: 10 μl of anti-CD133 conjugated with allo-phycocyanin (APC) (Milteny Biotec), 5 μl of anti-CD45 conjugated with APC-H7 (Becton Dickinson), 10 μl of anti-KDR (also known as type 2 vascular endothelial-growth factor receptor-VEGF-R2) conjugated with phycoerythrin (PE) (Sigma), 10 μl of anti-CD34 conjugated with fluorescein isothiocyanate (FITC) (Becton Dickinson) and 10 μl of anti-CD184 (also known as C-X-C chemokine receptor type 4 (CXCR4)) conjugated with PE-Cyanine 5 (PE-Cy5) (BD-Pharmingen) for 30 min at 4 °C, protected from light. Red blood cell lysis was performed using a 1:10 (v/v) water dilution of FACS Lysing Solution (BD-Biosciences) and washed with phosphate-buffered-saline (PBS) before flow-cytometry acquisition. Data acquisition was performed with a BD-Bioscience FACSCanto II, a high-performance flow cytometer, which can accurately analyse up to eight different fluorescent markers from many events. We used the flow cytometry software Infinicyt 1.5 (Cytognos) for the analysis. According to the Schmidt-Lucke’s and co-workers protocol, human circulating EPCs are identified by a minimal antigenic profile that includes at least one marker of stemness/immaturity (CD34 and/or CD133) and at least one marker of endothelial commitment (KDR) (see details in Supplementary Material). CD45 staining was also performed to identify and consequently exclude leucocytes (see details in Supplementary Material). In fact, as previously summarized^25,26^, among these only CD45^dim^ cells are considered circulating EPCs. In addition, we analysed CXCR4 (CD184), receptor of Stromal cell-derived factor 1 (SDF1), a cell surface antigen expressed on EPCs that plays a key role in their trans-endothelial migration and homing to sites of vascular injury. However, since isotype controls are known to mask rare cell populations, none were used in this analysis, and baseline fluorescence was determined using unstained cells. Moreover, EPCs are extremely rare events in peripheral blood, and thus require additional strategies to increase the sensitivity of the method and the accuracy of our work. These included: the automatic compensation to minimize fluorescence spillover, the exclusion of dead cells and the use of specific high-quality mononuclear antibodies^25–27^. The total number of acquired events was increased to a minimum of 1 million per sample, which is several folds larger than the number of events required for most other applications of flow cytometry. Thus, the results of counts from flow cytometry were expressed as the number of cells per 10^6^, while standard deviations (SD) were reported beside the mean of each group. Circulating EPCs counts, performed in triplicate, were tested by a non-parametrical Spearman test, showing a strong statistical correlation (r = 0.83, *P* < 0.0001). These technological approaches were used for all the enrolled groups. In conclusion, we quantified four different populations of EPCs, similarly to Schmidt-Lucke and co-workers: (1) CD45^dim^CD133^+^KDR^+^cells; (2) CD45^dim^CD34^+^KDR^+^cells; (3) CD45^dim^CD34^+^CD133^+^KDR^+^triple positive cells; and (4) the subpopulation of CD45^dim^CD34^+^KDR^+^CXCR4^+^ EPCs.

### Quantifications of systemic levels of Notch-1, VEGF, SDF-1 (CXCL12)

Venous blood samples were collected from all enrolled subjects in a fasting state (more than 8 h without food administration). In BAV and TAV cases, blood samples were collected within the first 3 h of their admission. Plasma samples were obtained immediately after collection spinning whole blood (3500 rpm at 4 °C for 10 minutes) and then stored at −80 °C for further analysis. Plasma levels of VEGF and SDF-1 were measured by ELISA and commercial kits (R&D Systems, Minneapolis, MN, USA), according to the manufacturer’s instructions. Notch-1 systemic levels were also detected using an ELISA test (Cusabio, Baltimore, MD, USA). Precisely, we detected the soluble form of Notch-1 pathway, which represents the Notch Extracellular Domain (*see Results and Discussion sections for its detailed description*). Detection limits were 9 pg/ml, 3.9 pg/ml and 0.01 pg/ml for VEGF, SDF-1 and Notch-1 respectively. All assays were run in duplicate.

### Transcription analyses by using Real-time PCR (qRT-PCR)

Full aortic segments with resected normal as well as aneurysmatic aortic wall from tubular-ascending aorta were collected from patients with AAA. Specimens were fixed in 10% neutral buffered formalin and then processed for routine paraffin embedding. Total RNA was extracted by using the QiagenRNeasy FFPE kit, treated with DNase I enzyme (Promega) for 1 h at 37 °C and then cleaned by column purification (Qiagen). Total RNA concentration and quality were determined with a spectrophotometer. Then cDNA was prepared using 1–2 µg of RNA (Ready-To-Go, T-Primed First-Strand Kit, Amersham Bioscience). The synthesised cDNA was stored at −20 °C RT-PCR analysis. Quantitative polymerase chain reaction (qPCR) was subsequently performed using the SYBR Green (SIGMA) in a Light-Cycler (Roche). Cyclophilin and Rn18S transcripts were used as internal controls^36^. Primer sequences are illustrated in Table S1 Supplementary Materials. The results were analysed using the 2^−ΔΔCt^ (Livak) relative expression method.

### Statistical analysis

Statistical analyses were performed using SPSS software version 20. Significant differences among qualitative variables were calculated by using *χ*2 test. Continuous variables (including number of cells, levels of systemic blood molecules, and gene expression levels) were expressed as mean ± SD (Standard deviation). Distribution normality was assessed by Kolmogorov–Smirnov test. Unpaired *t*-test (Welch corrected) was utilised to analyse the data between two groups whereas one-way ANOVA or Kruskal-Wallis test followed by Bonferroni correction was applied to compare more than two groups. To identify possible correlations, a non-parametrical Spearman correlation test was also used. To determine the possible influence of Notch-1 on the EPC populations, we used a generalized linear model with Poisson distribution and logarithm link. Differences were considered significant when a p value < 0.05 was obtained by comparison between the different groups.

## Electronic supplementary material

Supplementary Information
