# Supplementary material for: Deregulation of Notch1 pathway and circulating endothelial progenitor cell (EPC) number in patients with bicuspid aortic valve with and without ascending aorta aneurysm
Source: Sci Rep. 2018 Sep 14;8:13834. doi: 10.1038/s41598-018-32170-2 (PMC6138685; doi:10.1038/s41598-018-32170-2)
Supplement: Supplementary file 1 — Supplementary Information [file 41598_2018_32170_MOESM1_ESM.doc]

**Supplementary material:**

**Deregulation of Notch1 pathway and circulating endothelial progenitor cells (EPCs) number in patients with bicuspid aortic valve with and without ascending aorta aneurysm**

**Carmela R. Balistreri^1^, Floriana Crapanzano^1^, Leonardo Schirone^2^, Alberto Allegra^3^, Calogera Pisano,^4^ Giovanni Ruvolo^4^, Maurizio Forte^5^, Ernesto Greco,^6^ Elena Cavarretta^2^, Antonino G.M. Marullo ^2^, Sebastiano Sciarretta^2,5^ & Giacomo Frati^2,5^**

^1^Department of Pathobiology and Medical Biotechnologies, University of Palermo, Palermo, Italy. ^2^Department of Medico-Surgical Sciences and Biotechnologies, Sapienza University of Rome, Latina, Italy. ^3^Department of Surgery and Oncology, University of Palermo, Italy. ^4^Department of Experimental Medicine and Surgery, University of Rome Tor Vergata, Rome, Italy. ^5^IRCCS NEUROMED, Pozzilli (IS), Italy. ^6^Department of Cardiovascular, Respiratory, Nephrological, Anesthesiological, and Geriatric Sciences, Sapienza University of Rome, Rome, Italy.

Correspondence and requests for materials should be addressed to C.R.B. (email: carmelarita.balistreri@unipa.it)

**Schmidt-Lucke and co-workers s’ standardized protocol according to the modified sequential gating strategy of International Society for Hematotherapy and Graft Engineering (ISHAGE)** (*see references n. 33,34 in the text*)

As reported in the text, for quantifying the circulating EPC cells the Schmidt-Lucke and co-workers s’ standardized protocol *(see reference n.33 and 34 of the text*), derived by the sequential gating strategy of ISHAGE, was used. Briefly, we reported the gating strategy of ISHAGE modified for the first time by Schmidt-Lucke and co-workers s’ and reproduced in our study. It consisted, as shown in Figure 1 of the study by Schmidt-Lucke and co-workers, in an initial gate (R1 plot 1) set on a CD45 vs. side scatter (SSC) dot plot to contain all CD45^+^ events, including CD45^dim^ and CD45^bright^. This permitted to exclude CD45^−^ events (i.e. red blood cells, platelets and other debris). The lower limit of CD45 expression was adapted from a CD34 vs. CD45 histogram performed on ungated data (plot 5). R1 also contained a gate R4, which defined lymphocytes as CD45^bright^SSC^low^ cells. The events in gate R1 were then displayed on a CD34 vs. SSC dot plot (plot 2) and a second gate (R2) was produced to include the cluster of CD34^+^ events. The third plot (plot 3) was obtained by plotting the events that fulfil the criteria of gates R1 and R2 (i.e. sequential gating). Cells forming a cluster of blasts with characteristic low SSC and low CD45 fluorescence (SSC^low^CD45^dim^ cells) were then gated on this third plot to produce a third region (R3) (plot 3). To differentiate between CD45^dim^ and CD45^bright^ cells, the right margin of the CD34^+^ cells in plot 5 was used as cut-off. At this step, the gating strategy defined CD34^+^CD45^dim^ cells. In case, CD45^bright^ cells were investigated for comparison, gate R3 was shifted to the right population visible in plot 3. Finally, the events fulfilling the criteria of all three gates (R1, R2 and R3) were then displayed on a forward light scatter (FSC) vs. SSC dot plot to confirm that the selected blasts fall into the lymphocyte region (R5) (plot 4). The lymphocyte region (plot 6) was adjusted from a SSC vs. FSC plot gated on lymphocytes from R4 (plot 1), employing only small lymphocytes (FSC^low^, R5 in plot 6). CD45^dim^CD34^+^KDR^+^ endothelial progenitor cells were then deducted from the upper right quadrant of plot 7, defining CD34^+^KDR^+^ cells, when falling into all three regions (R2, R3 and R5). Isotype controls were not required because the gating strategy used excluded cells that non-specifically bind anti-CD34.

**Table S1. Primer sequences for qRT-PCR**

| **Human gene** | **Forward** | **Reverse** |
| --- | --- | --- |
| **Notch-1** | **5’ ACTGTGAGGACCTGGTGGAC 3’** | **5’ TTGTAGGTGTTGGGGAGGTC 3’** |
| **Notch-2** | **5’ AAGCAGAGTCCCAGTGCCTA 3’** | **5’ CAGGGGGCACTGACAGTAAT 3’** |
| **Notch-3** | **5’ TGTGGACGAGTGCTCTATCG 3’** | **5’ AATGTCCACCTCGCAATAGG 3’** |
| **Notch-4** | **5’ CTAGGGGCTCTTCTCGTCCT 3’** | **5’ CAACTTCTGCCTTTGGCTTC 3’** |
| **Jagged-1** | **5’ GACTCATCAGCCGTGTCTCA 3’** | **5’ TGGGGAACACTCACACTCAA 3’** |
| **Jagged-2** | **5’-TGGGCGGCAACTCCTTCTA-3’** | **5’ GCCTCCACGATGAGGGTAAA3’** |
| **DLL1** | **5’GATTCTCCTGATGACCTCGCA 3’** | **5’TCCGTAGTAGTGTTCGTCACA 3’** |
| **DLL3** | **5’CACTCCCGGATGCACTCAAC3’** | **5’GATTCCAATCTACGGACGAGC3’** |
| **DLL4** | **5’ GTCTCCACGCCGGTATTGG3’** | **5’CAGGTGAAATTGAAGGGCAGT3’** |
| **Hes-1** | **5’ CTCTCTTCCCTCCGGACTCT 3’** | **5’ AGGCGCAATCCAATATGAAC 3’** |
| **Hey-1** | **5’ CGAGGTGGAGAAGGAGAGTG 3’** | **5’ CTGGGTACCAGCCTTCTCAG 3’** |
| **Hey-2** | **5’ GAACAATTACTCGGGGCAAA 3’** | **5’ TCAAAAGCAGTTGGCACAAG 3’** |
| **NRARP** | **5’ GACTCAATTCGAACCCGAAA 3’** | **5’ ACTTCCATGAAGGGGAAACC 3’** |
| **Cyclophilin** | **5’ TTCATCTGCACTGCCAAGAC 3’** | **5’ TCGAGTTGTCCACAGTCAGC 3’** |
| **Rn18S** | **5’ AAACGGCTACCACATCCAAG 3’** | **5’ CCTCCAATGGATCCTCGTTA 3’** |
